# Spontaneous Differentiation of T Follicular Helper Cells in LATY136F Mutant Mice

**DOI:** 10.3389/fimmu.2021.656817

**Published:** 2021-04-12

**Authors:** Sarah A. O’Brien, Minghua Zhu, Weiguo Zhang

**Affiliations:** ^1^ Department of Immunology, Duke University Medical Center, Durham, NC, United States; ^2^ Suzhou Institute of Systems Medicine, Chinese Academy of Medical Sciences, Suzhou, China

**Keywords:** T follicular helper cells, Th2 differentiation, cytokine production, TCR signaling, adaptor protein

## Abstract

Mice with a mutation at the LAT-PLCγ1 binding site (Y136) have a defect in thymocyte development due to dampened TCR signaling. CD4^+^ T cells that do reach the periphery are hyper-activated and skewed to Th2. Over time, these mice develop an autoimmune-like syndrome, characterize by overproduction of Th2 cytokines, T cell infiltration into various organs, and B cell activation, isotype switching, and autoantibody production. In this study, we examined IL4 production by CD4^+^ T cells in the LATY136F mice using the KN2 reporter mice, in which human CD2 expression marks T cells that are actively producing IL4 protein. We showed that these mice had spontaneous Tfh differentiation. Despite the fact that the majority of CD4^+^ T cells were skewed to Th2 and were GATA3^+^, only a small subset of them were actively secreting IL4. These T cells were Tfh cells that expressed BCL6 and were localized to B cell-rich germinal centers within the spleen. Interestingly, these Tfh cells expressed high levels of both BCL6 and GATA3. By using LAT conditional knockout mice that inducibly express only the LATY136F allele, we further showed that Tfh cell differentiation was likely the result of defective LAT-PLCγ1 signaling in the periphery. In addition, B cells were required for spontaneous development of Tfh cells and uncontrolled T cell expansion in these mice. Together, these results indicated a novel role for tonic LAT-PLCγ1 signaling in modulating Tfh cell differentiation during development of autoimmune syndrome.

## Introduction

Th1 T cells play important roles in type 1 immune responses against intracellular bacteria and viruses by secreting IFNγ and promoting CD8^+^ T cell cytotoxicity. On the other hand, Th2 cells function in type 2 immune responses against extracellular pathogens, such as helminth, by inducing B cell activation and IgE secretion, resulting in tissue eosinophilia, mast cell activation, and macrophage activation (1). Type 2 immunity requires IL4 and IL13, two cytokines that are secreted by T cells and other cell types.

T follicular helper cells (Tfh) are a subset of CD4^+^ effector T cells that help B cells within the germinal center, where antibody affinity maturation and isotype switching occur (2). Previous studies indicate that during allergic inflammation, IL4 in lymphoid tissues is mainly produced by Tfh cells, not by Th2 cells (3). Tfh cells express CXCR5, which is the receptor for CXCL13 (4–6). CXCL13 is a chemokine produced by follicular stroma cells (7, 8). CXCR5 expression promotes T cell migration into the follicles. BCL6 is a transcription factor vital for Tfh differentiation (9, 10). Interestingly, it also functions as a transcriptional repressor to block expression of other transcription factors, such as GATA3, T-bet, and RORγt (10). These transcription factors are important for differentiation of other helper T cell subsets.

LAT is an adaptor molecule that is phosphorylated by ZAP-70 tyrosine kinase after T cell receptor (TCR) engagement (11). Through binding to Grb2, Gads, and PLC-γ1, it mediates activation of multiple downstream signaling pathway (12). Previous studies have clearly demonstrated that LAT is required for TCR-mediated signaling, T cell activation, and thymocyte development (13, 14). LATY136F mice, which have a mutation at the LAT binding site for PLC-γ1, display a spontaneous Th2-type autoimmune syndrome (15, 16). CD4^+^ T cells in the spleens and lymph nodes of these mice produce large amounts of Th2 cytokines, such as IL4 and IL13. They are also hyperproliferative, resulting in enlarged secondary lymphoid organs. Additionally, these T cells infiltrate into the liver, lung, and kidney of these mice. As a result of the abnormal T cell activation, B cells become activated and produce high concentrations of serum IgE and IgG1 (15, 16).

Several studies have been done to explore various aspects of T cell development and peripheral function to better understand the cause of LATY136F-mediated autoimmunity. One study using the HY transgenic TCR mice indicated that LATY136F mice have defects in both positive and negative thymic selection (17), and mutant CD4^+^ T cells display self-reactivity (16). In addition, the development of regulatory T cells is impaired as the LATY136F mice lacking detectable thymic and peripheral CD4^+^FOXP3^+^ T cells (18). To further study T cell hyperproliferation in these mice, we developed an inducible system (ERCre^+^LAT^f/m^, m=Y136F) in which the wild-type floxed LAT (f) allowed for proper T cell thymic development and then could be deleted by tamoxifen treatment in peripheral T cells, resulting in expression of the LATY136F allele only. Although Tregs were present in the spleens and lymph nodes of these mice, they failed to suppress conventional CD4^+^ T cell proliferation, indicating that the LAT-PLCγ1 interaction is also required for Treg function. T cells in tamoxifen-treated mice are skewed to Th2 and cause a similar autoimmune syndrome, indicating that it is the aberrant LAT-PLCγ1 signaling in mature T cells that drives Th2 skewing *in vivo* (19). In addition, our data also showed that IL6 is involved in T cell hyperproliferation in these mice as IL6 deficiency blocks uncontrolled T cell expansion during the early stage of disease development. The mutant T cells overproduce IL6 due to activated NF-κB, AKT, and p38 pathways (20). Taken together, these data indicate that the LAT-PLC-γ1 interaction is important in maintaining T cell homeostasis through regulating development and function of Treg cells and controlling differentiation through production of cytokines.

Given the known role for LAT-PLCγ1 signaling in cytokine production by CD4^+^ T cells (15, 16, 20, 21), in this study we would like to understand the role of this signaling pathway in Tfh cell development. Since the LATY136F CD4^+^ T cells produce a large amount of IL4, we wanted to identify which subset of T cells actively produce IL4 *in vivo* by using IL4 reporter mice. Our data indicated that LAT-PLCγ1 signaling plays an important role in Tfh cell lineage commitment and functions to control the development of autoimmune diseases.

## Materials and Methods

### Mice

LATY136F (LAT^m/m^), LAT^-/-^, and ERCre^+^LAT^f/f^ mice have been previously described (14, 15, 22). IL4 reporter mice (IL4^KN2^) were kindly provided by Dr. R. Lee Reinhardt (Duke University, Durham, NC). These mice were crossed with LAT^m/m^ to generate LAT^m/m^IL4^KN2/+^ mice. ICOS^-/-^ and RAG2^-/-^ mice were purchased from the Jackson Laboratory (Bar Harbor, ME). All mice used were on a C57Bl/6 background. Mice were housed in specific pathogen-free conditions and were used in accordance with the National Institutes of Health guidelines. All experiments were approved by the Duke University IACUC.

### Flow Cytometry

Single cell suspensions from spleens or lymph nodes were stained with various antibodies (BioLegend) in the presence of 2.4G2 (anti-FcγII/III receptor). The anti-human CD2 antibody was from Invitrogen. Intracellular staining for GATA3 and BCL6 was performed after fixation and permeabilization using a kit from eBioscience. Data were acquired on the FACSCanto II (BD Bioscience) and analyzed using the FlowJo software.

### Real-Time PCR

Total RNAs from purified CD4^+^ T cells were isolated using the Trizol reagent (Invitrogen). The SuperScript reverse transcriptase (Invitrogen) was used to synthesize cDNAs. The real-time PCR was done using SYBR Green supermix (Bio-Rad) to quantify cytokine RNAs.

### Immunofluorescence Imaging

Spleens were embedded in Tissue-Tek OCT compound (Sakura Finetek) and cut into 5μm sections on a Leica CM3050 cryomicrotome (Leica Microsystems). Sections were fixed with 1:1 acetone/methanol and huCD2-biotin was amplified using fluorescein-tyramide according to the TSA Fluorescein System protocol (PerkinElmer). Conjugated antibodies were used at 1:200 followed by 1:2000 dilution of 1mg/mL DAPI (Invitrogen), mounted using Fluoromount-G (SouthernBiotech), and analyzed on the SP5 confocal microscope (Leica).

### Tamoxifen Treatment

Tamoxifen was dissolved in corn oil at a concentration of 10mg/ml. 1.5mg/25g body weight was injected intraperitoneally into mice for two consecutive days for initial deletion of floxed *lat* alleles. For long-term deletion, the same dose was administered once a week. When *lat* was deleted in germline ERCre^+^LAT^f/+^ or ERCre^+^LAT^f/m^ mice, tamoxifen was injected at Days 0, 1, 7, and 14 with a takedown at Day 15 for flow cytometry analysis. For adoptive transfer studies, CD4^+^ T cells were transferred on Day 0 and tamoxifen was injected at Day 21, 22, 28, and 35 with a takedown at Day 36.

### T Cell Transfer Experiments

1x10^6^ ERCre^+^LAT^f/+^ or ERCre^+^LAT^f/m^ CD4^+^ T cells were sorted and injected intravenously into LAT^-/-^ or RAG^-/-^ mice. Three weeks after adoptive transfer, tamoxifen was injected intraperitoneally weekly for 3 weeks before the mice were sacrificed at 6 weeks after initial adoptive transfer. 2x10^6^ IL4^KN2/+^LAT^m/+^ or IL4^KN2/+^LAT^m/m^ CD4^+^ T cells were sorted and injected intravenously into LAT^-/-^ or RAG2^-/-^ mice. Mice were sacrificed 8 weeks later. Similarly, 2x10^6^ huCD2^-^ and huCD2^+^ CD4^+^ T cells from IL4^KN2/+^LAT^m/m^ mice were sorted and injected intravenously into LAT^-/-^ hosts. Mice were sacrificed 8 weeks later for flow cytometry analysis.

### Statistics

All studies were repeated 2-5 times, with an average of 5 mice per group. Flow cytometry data were graphed in GraphPad Prism and are shown as mean + standard deviation. Statistics were analyzed using unpaired, two-tailed T test or one-way ANOVA with Tukey’s multiple comparisons.

## Results

### IL4-Producing T Cells Are Localized in GC-Like Zones in LATY136F Mice

Published data indicate that LATY136F (LAT^m/m^) CD4^+^ T cells spontaneously develop a Th2 phenotype. Up to 90% of T cells produce IL4 after stimulation *in vitro* with PMA and ionomycin for four hours (15, 16). To monitor IL4 production in T cells *in vivo*, we crossed LAT^m/m^ mice with the KN2 reporter mice. In these reporter mice, the human CD2 gene (huCD2) was placed at the start site of the IL4 gene by homologous recombination. Thus, IL4-producing cells also express huCD2 on the cell surface, which can be easily detected by flow cytometry (23).

**Figure 1:**
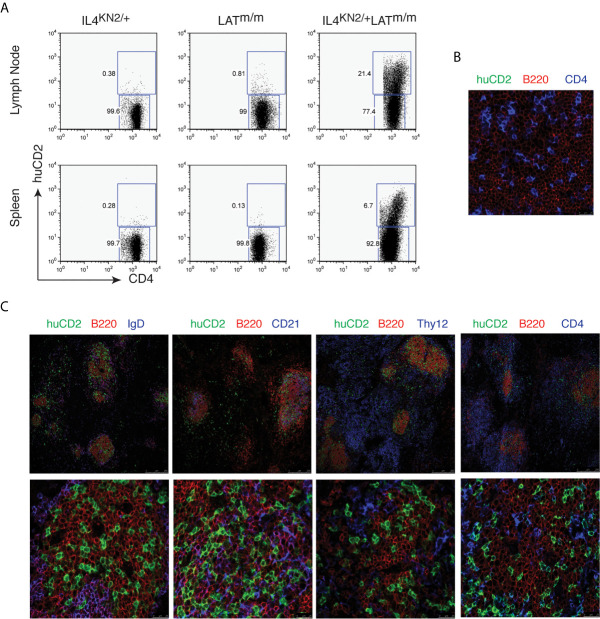
IL4-producing T cells in LATY136F mice. **(A)** IL4 production, indicated by human CD2 expression, in CD4^+^ T cells from spleens and inguinal lymph nodes from 6-8wk-old IL4^KN2/+^, LAT^m/m^, and IL4^KN2/+^LAT^m/m^ mice. **(B, C)** 5 μm spleen sections from 3-month-old LAT^m/m^ **(B)** and IL4^KN2/+^LAT^m/m^ **(C)** mice were stained with anti-mouse B220-PE (red), huCD2-biotin, amplified using fluorescein-tyramide (green), and biotinylated anti-CD4, IgD, CD21, and Thy1.2 antibodies followed by streptavidin Alexa Fluor 647 (blue). Imaging was performed using confocal microscopy at 20x (top) and 70x (bottom) magnification. Data are representative of 3 individual experiments.

We examined 6–8-week-old IL4^KN2/+^ and IL4^KN2/+^LAT^m/m^ mice for IL4 production in T cells from the lymph nodes and spleens by flow cytometry. While very few T cells produced IL4 in IL4^KN2/+^ mice and LAT^m/m^ mice without the KN2 reporter (0.38% and 0.81% in the inguinal lymph nodes and 0.28% and 0.13% in the spleens), 21.4% of CD4^+^ T cells in lymph nodes and 6.7% in the spleens of IL4^KN2/+^LAT^m/m^ mice were actively producing IL4 (**Figure 1A**). It was unexpected to detect a much smaller percentage of these CD4^+^ T cells producing IL4 *in vivo*, considering *nearly* all LAT^m/m^ T cells produced IL4 upon PMA and ionomycin stimulation *in vitro* (data not shown). Next, we examined where these IL4-producing T cells were localized in the secondary lymphoid tissues. Spleen sections were stained with anti-human CD2 and antibodies to identify T (CD4, Thy1.2) and B (B220, IgD, and CD21) cells and imaged on a SP5 confocal microscope. Previously, it was reported that LAT^m/m^ mice have spontaneous germinal center formation (24). We used LAT^m/m^IL4^+/+^ mice as negative controls for huCD2 staining. As expected, CD4^+^ T cells were clearly found within the B cell-rich regions of the spleen, and none of them were huCD2^+^ (**Figure 1B**). In LAT^m/m^IL4^KN2/+^ mice, CD4^+^ T cells that were clustered in the B220^+^ B cell rich regions of the spleen were huCD2^+^, indicating that they were actively producing IL4 (**Figure 1C**). In the area outside of the B cell zone, only a small number of huCD2^+^ T cells were found. Further staining showed that most of these B cells in the germinal center were IgD^-^, indicating that these B cells had undergone class switch recombination. CD21 is expressed on mature B cells and follicular dendritic cells. CD21 was also colocalized with those IL4-producing LAT^m/m^ T cells (**Figure 1C**). Thus, while the architecture of LAT^m/m^ secondary lymphoid organs was abnormal, IL4-producing T cells were localized to the germinal center-like regions and were in close contact with B cells that had undergone isotype-switching.

### IL4-Producing T Cells Are T Follicular Helper Cells

**Figure 2:**
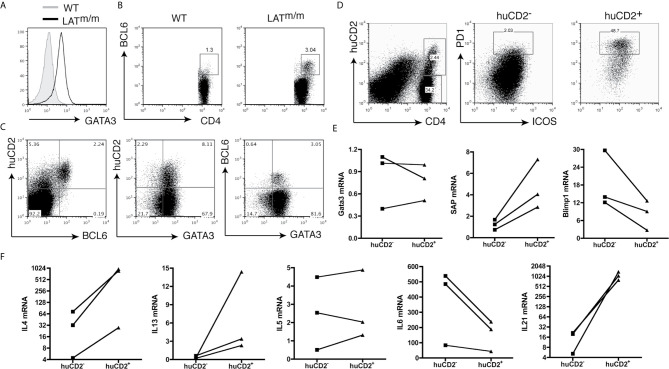
IL4-producing CD4^+^ T cells in LATY136F mice are Tfh cells. **(A)** Intracellular staining of GATA3. Cells were pre-gated on splenic CD4^+^ T cells. **(B)** BCL6 expression in CD4^+^ T cells from the spleens of WT and LAT^m/m^ mice. **(C)** Flow cytometry analysis of huCD2, GATA3, and BCL6 expression on CD4^+^ T cells from IL4^KN2/+^LAT^m/m^ mice. **(D)** ICOS and PD1 expression on huCD2^+^ and huCD2^-^ CD4^+^ T cells from IL4^KN2/+^LAT^m/m^ mice (6-8 weeks old). **(E, F)** Real-time PCR analysis. RNA levels of GATA3, SAP, Blimp1, and different cytokines from sorted huCD2^+^ and huCD2^-^ CD4^+^ T cells from 3-month-old IL4^KN2/+^LAT^m/m^ mice were normalized to β-actin. Connected lines indicate sorted populations from the same mouse. Data are representative of 2-5 independent experiments.

We previously showed that the LAT-PLCγ1 interaction is important in Th2 differentiation. Even γδ LATY136F T cells in secondary lymphoid organs of these mice express high levels of GATA3 and produce IL4 *in vitro (* 25). Based on our imaging data, T cells that were actively producing IL4 in LAT^m/m^ mice were localized to the germinal centers. During immune responses, IL4 in secondary lymphoid organs is mainly produced by follicular helper T cells (Tfh) (3). Thus, these LAT^m/m^ IL4-producing cells were likely Tfh cells, rather than Th2 cells. To determine whether these cells were indeed Tfh cells, we stained splenocytes with antibodies against proteins that are commonly expressed in Tfh cells. In support of our previous findings, we observed that nearly all LAT^m/m^ CD4^+^ T cells expressed high levels of GATA3, the master regulator for Th2 cells (**Figure 2A**). This might explain why the majority of these CD4^+^ T cells were IL4 competent and could produce IL4 after PMA and ionomycin stimulation *in vitro*. Interestingly, when we stained for BCL6, the master regulator for Tfh cells, we observed that ~3% of CD4^+^ T cells in the spleen were BCL6^+^ (**Figure 2B**). We further analyzed BCL6 and GATA3 expression in huCD2^+^ cells. As shown in **Figure 2C**, nearly all BCL6^+^ T cells were huCD2^+^ and thus were IL4 producers, while the majority of CD4^+^ T cells in the spleen were GATA3^+^ but did not produce IL4. In addition, there were huCD2^low^ cells that were BCL6^-^ (**Figure 2C**). Our data indicated that within the secondary lymphoid organs of LAT^m/m^ mice, the majority of CD4^+^ T cells were skewed to Th2 and were GATA3^+^BCL6^-^. They only produced IL4 and IL13 after *in vitro* stimulation. A small percentage of T cells were BCL6^+^ and were actively producing IL4 *in vivo*, although they also expressed GATA3.

It is known that BCL6 suppresses GATA3 expression at the post-transcriptional level (9, 26). Previous studies on Tfh cells in the lymph nodes after *N. brasiliensis* infection indicate that BCL6^+^ T cells do not express GATA3 (27). Remarkably, in LAT^m/m^ T cells, both huCD2^+^ and huCD2^-^ cells showed equally high levels of GATA3. The same was also true with BCL6^+^ and BCL6^-^ T cells (**Figure 2C**). To confirm this result, we further sorted huCD2^+^ and huCD2^-^ CD4^+^ T cells from LAT^m/m^ mice by FACS and performed RT-PCR analysis. In agreement with the intracellular staining data, huCD2^+^ and huCD2^-^ cells indeed had similar GATA3 mRNA expression (**Figure 2E**).

To confirm that huCD2^+^BCL6^+^ T cells in LATY136F mice were indeed Tfh cells, we also compared the expression of other Tfh signature proteins in huCD2^+^ and huCD2^-^ populations. Tfh cells express high levels of PD1, ICOS, CXCR5, SAP, and IL21 (3, 27), and low levels of Blimp1, a BCL6 antagonist (28). Surface staining of T cells from IL4^KN2/+^LAT^m/m^ mice revealed that huCD2^-^ T cells had very few ICOS^+^PD1^+^ cells (~2%), while huCD2^+^CD4^+^ T cells were enriched for this population (~49%) (**Figure 2D**). T cells expressing huCD2 also expressed more SAP and less Blimp1 compared to the huCD2^-^ counterparts (**Figure 2E**).

Tfh cells can produce various T helper cytokines, such as IFNγ, IL4, IL5, IL13, and IL17 (29). Additionally, IL6 is potentially important for their differentiation (10, 30–32). Since LATY136F mice have spontaneous type 2 immune responses, and our previous work shows that T cells from these mice overproduce IL6 (20), we examined the levels of these cytokines in huCD2^+^ and huCD2^-^ LAT^m/m^ T cells. As shown in **Figure 2F**, IL4, IL13, and IL21 levels were higher in huCD2^+^ T cells than in huCD2^-^ T cells, while IL6 was lower. IL5 expression was similar in these two populations. These data indicated that this huCD2^+^ population is phenotypically distinct from Th2 cells. These results were similar to those using *Leishmania major* or *Nippostrongylus brasiliensis* (3). During infection by these pathogens, IL4 production within the secondary lymphoid organs is restricted to Tfh cells, rather than Th2 cells. Our data suggested that LAT-PLCγ1 signaling could modulate Tfh cell development.

### Defective LAT Signaling in the Periphery Drives Spontaneous Tfh Differentiation

Both positive and negative selection are impaired in LAT^m/m^ mice (17), which may lead to generation of high-affinity autoreactive T cells. Previous studies have suggested that during differentiation, T cells with high TCR avidity preferentially develop into Tfh over non-Tfh cells (33), which might be amplified in a setting where thymocyte selection is abnormal. Therefore, we wanted to understand if spontaneous Tfh cell differentiation in these mice was a result of altered LAT-PLCγ1 signaling during T cell development in the thymus or in the periphery.

To answer this question, we used our inducible ERCre^+^LAT^f/m^ system, where a wildtype floxed LAT allele (f) allows normal thymocyte development, but then can be deleted in the periphery by injection of tamoxifen, leaving only the LATY136F allele (19). In this system, two LoxP sites flank exons 7-11, and deletion of these exons not only leads to a non-functional LAT allele, but also expression of GFP. After tamoxifen treatment for 4 weeks, ERCre^+^LAT^f/m^ mice showed similar CD4^+^ T cell hyperproliferation, IL4 production, and GATA3 expression as LATY136F mice (19, 34).

**Figure 3:**
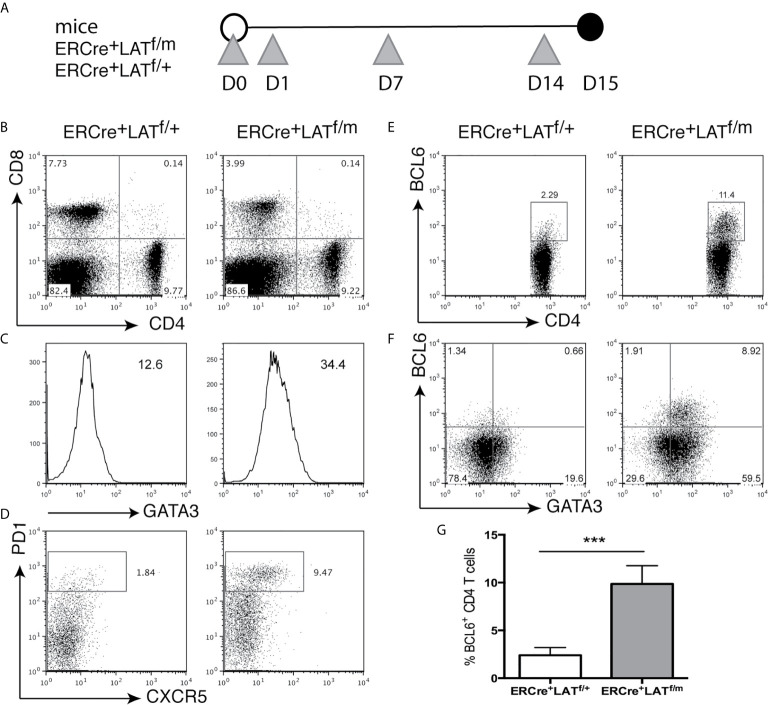
Tfh differentiation in ERCre^+^LAT^f/m^ mice. **(A)** ERCre^+^LAT^f/+^ and ERCre^+^LAT^f/m^ mice were injected at day 0, 1, 7, and 14 with tamoxifen prior to being sacrificed on day 15. Grey triangles indicate the day of tamoxifen treatment. **(B)** CD4 and CD8 expression in the spleen. **(C)** Intracellular staining of GATA3 in CD4^+^ T cells. Numbers indicate gMFI. **(D)** Cells were pre-gated on CD4^+^ for CXCR5 vs. PD1 expression. **(E)** Intracellular staining of BCL6 in CD4^+^ T cells. **(F)** GATA3 and BCL6 expression in CD4^+^ T cells. **(G)** The percentage of CD4^+^ T cells expressing BCL6. Data are representative of 2 individual experiments. Two-tailed t test; ***p < 0.001.

To assess Tfh cell development prior to B cell activation, we examined ERCre^+^LAT^f/+^ and ERCre^+^LAT^f/m^ mice 2 weeks after tamoxifen injections at Day 0, 1, 7, and 14 (**Figure 3A**). All T cells were GFP^+^ (not shown), indicating that the wildtype LAT allele was efficiently deleted in these cells; yet the CD4^+^ T cell hyperproliferative disease development had not yet begun to occur. ERCre^+^LAT^f/m^ mice did not have enlarged spleens and lymph nodes and had similar percentages of CD4^+^ T cells compared to ERCre^+^LAT^f/+^ mice (data not shown and **Figure 3B**). Interestingly, at this early time point, we saw that ERCre^+^LAT^f/m^ CD4^+^ T cells had already upregulated GATA3 expression (**Figure 3C**). When we examined these mice for Tfh cells, we saw an increased percentage of PD1^+^CXCR5^+^ in the CD4^+^ T cell population (~9.5% for ERCre^+^LAT^f/m^ vs. 1.8% for ERCre^+^LAT^f/+^, **Figure 3D**). Correspondingly, there was a distinct population of BCL6^+^CD4^+^ T cells in the spleens of ERCre^+^LAT^f/m^ mice that was also GATA3 high (**Figures 3E, F**). The percentage of BCL6^+^ T cells in ERCre^+^LAT^f/m^ was significantly increased (**Figure 3G**). These data suggested that Tfh cell differentiated rapidly in peripheral T cells expressing the LATY136F mutation, but there was still a possibility that these Tfh cells were recent thymic emigrants in these mice.

**Figure 4:**
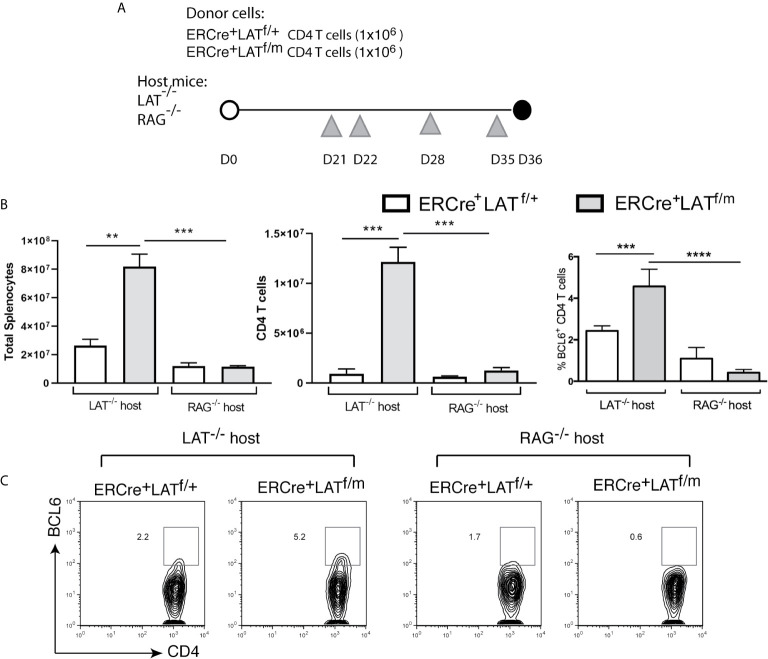
The importance of B cells in Tfh cell differentiation in LATY136F mice. **(A)** 1x10^6^ CD4^+^ T cells from ERCre^+^LAT^f/+^ or ERCre^+^LAT^f/m^ mice were sorted and transferred into LAT^-/-^ or RAG^-/-^ hosts. Three weeks later, tamoxifen was injected at day 21, 22, 28, and 35 before being sacrificed on day 36. Grey triangles indicate the day of tamoxifen treatment. **(B)** Cell counts. Total numbers of splenocytes, CD4^+^ T cells in the spleens, and the percentage of CD4 T cells expressing BCL6. **(C)** Intracellular staining for BCL6. Data are representative of 2-3 individual experiments. One-way ANOVA; Tukey’s multiple comparisons **p < 0.005; ***p < 0.001; ****p < 0.0001.

To eliminate the complication of recent thymic output in these mice after tamoxifen treatment and examine the role of B cells in spontaneous Tfh differentiation (see description of the results below), we sorted 1x10 (6) ERCre^+^LAT^f/+^ and ERCre^+^LAT^f/m^ CD4^+^ T cells and injected into LAT^-/-^ (T cell-deficient) and RAG2^-/-^ (T and B cell-deficient) mice. After the transfer, we waited 3 weeks to allow expansion by homeostatic proliferation and then injected tamoxifen at Day 21, 22, 28, and 35 to ensure complete deletion of the floxed LAT allele (**Figure 4A**). Among these mice, only the LAT^-/-^ mice receiving ERCre^+^LAT^f/m^ CD4^+^ T cells had enlarged spleens and lymph nodes (not shown) as reflected by increased numbers of splenocytes and CD4^+^ T cells (Fig.4B). There were more CD4^+^BCL6^+^ Tfh cells in LAT^-/-^ mice receiving ERCre^+^LAT^f/m^ T cells (5.2%) than those receiving ERCre^+^LAT^f/+^ T cells (2.2%, **Figures 4B, C**). These data suggested that spontaneous Tfh development is a consequence of aberrant LAT signaling, although we could not exclude the possibility that defective thymic selection could also contribute.

### B Cells Are Important for Tfh Cell Initiation and Maintenance in LATY136F Mice

The main function of Tfh cells is to help with B cell survival, class switching, and affinity maturation through the production of cytokines and expression of various surface co-receptors. To understand the requirement for B cells in the differentiation and maintenance of Tfh cells in LATY136F mice, we also performed adoptive transfer of ERCre^+^LAT^f/+^ and ERCre^+^LAT^f/m^ CD4^+^ T cells into RAG2^-/-^ mice (**Figure 4A**). As shown in **Figure 4B**, the absence of B cells in RAG^-/-^ hosts prevented splenomegaly and T cell expansion, which was only seen in LAT^-/-^ host upon transfer of ERCre^+^LAT^f/m^ CD4^+^ T cells (**Figure 4B**). These data implicated a role of B cell-T cell crosstalk in the hyperproliferative phenotype associated with LATY136F-mediated autoimmunity. We also assessed BCL6 expression in CD4^+^ T cells from these mice. While approximately 5% of CD4^+^ T cells in LAT^-/-^ mice received ERCre^+^LAT^f/m cells^ were BCL6^+^, only 0.6% of them were BCL6^+^ when transferred into RAG2^-/-^ hosts. In contrast, similar percentages of ERCre^+^LAT^f/+^ CD4^+^ T cells were BCL6^+^ in both LAT^-/-^ and RAG2^-/-^ hosts (**Figure 4C**).

**Figure 5:**
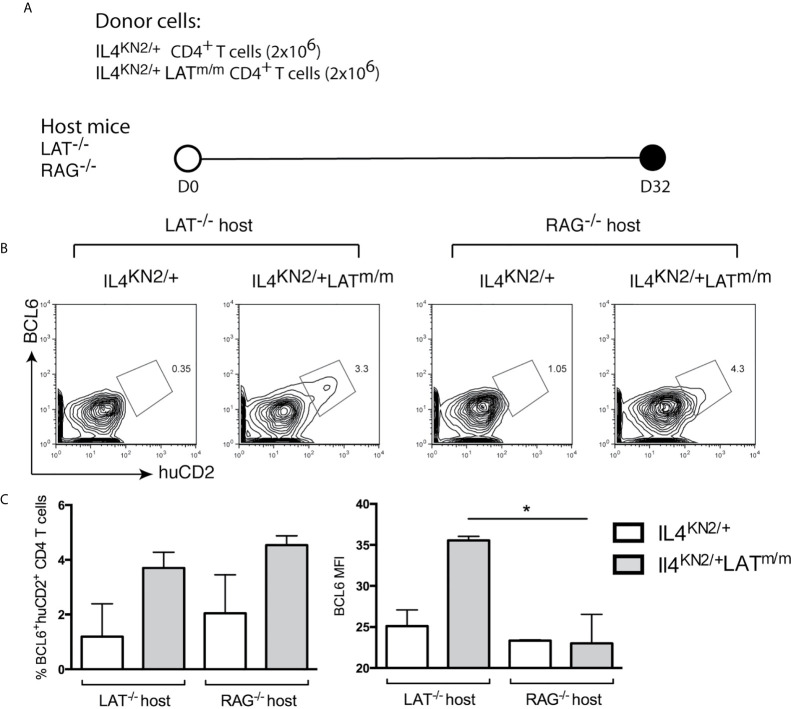
B cells are important for maintaining BCL6 expression in Tfh cells. **(A)** 2x10^6^ CD4^+^ T cells were sorted from 8 week-old IL4^KN2/+^ and IL4^KN2/+^LAT^m/m^ mice. Cells were i.v injected into LAT^-/-^ or RAG^-/-^ recipient mice for 8 wks. **(B)** BCL6 and huCD2 expression on CD4^+^Thy1.2^+^ transferred T cells. **(C)** The percent of BCL6^+^huCD2^+^ cells and intensity of BCL6 staining. Data are representative of 2-3 individual experiments. Two-tailed t test; *p < 0.05.

To examine the requirement of B cells in the maintenance of Tfh cells, we sorted CD4^+^ T cells from 8-week-old IL4^KN2/+^ and IL4^KN2/+^LAT^m/m^ mice and transferred them into both LAT^-/-^ and RAG2^-/-^ hosts (**Figure 5A**). Eight weeks after transfer, we assessed the persistence of a BCL6^+^ population of T cells in the spleens. Similar to germline IL4^KN2/+^LAT^m/m^ mice, the IL4 producing cells in mice with transferred IL4^KN2/+^LAT^m/m^ T cells were also BCL6^+^ (**Figure 5B**). Both LAT^-/-^ and RAG2^-/-^ mice that received IL4^KN2/+^LAT^m/m^ T cells had an average of ~4% huCD2^+^BCL6^+^ T cells; yet the overall intensity (MFI) of BCL6 in those T cells was significantly reduced in RAG2^-/-^ mice (**Figure 5C**). Together, these data suggested that B cells were important for spontaneous development and maintenance of Tfh cells and T cell hyperproliferation caused by abnormal LAT signaling.

### Tfh Lineage in LATY136F Mice Is Not Terminally Differentiated

**Figure 6:**
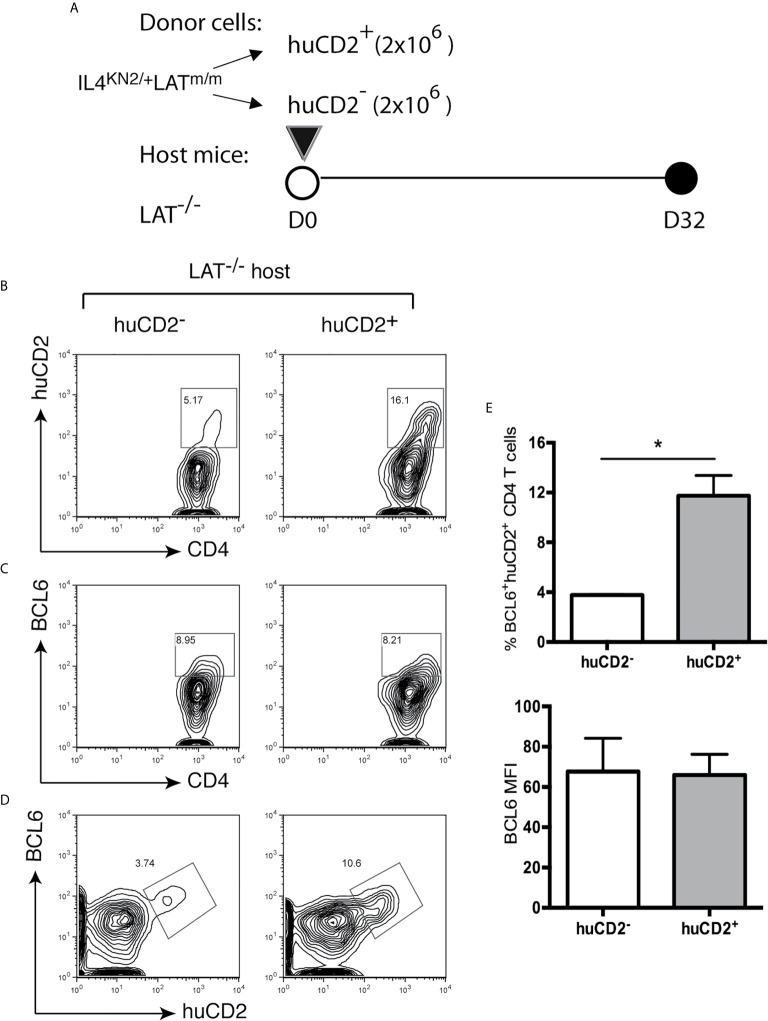
Instability of the LATY136F Tfh signature. **(A)** 1.5x10^6^ huCD2^+^ and huCD2^-^ CD4^+^ T cells were sorted from IL4^KN2+^LAT^m/m^ mice and transferred into LAT^-/-^ mice. Mice were sacrificed 8 weeks later and analyzed by FACS. CD4 and Thy1.2 were used to pre-gate on transferred T cells. **(B)** Representative flow plots of CD4 verse huCD2. **(C, D)** Intracellular staining for BCL6. **(E)** The percent of BCL6^+^huCD2^+^ cells of total CD4^+^ T cells and BCL6 gMFI pre-gated on BCL6^+^huCD2^+^CD4^+^ T cells. Two-tailed t test; *p < 0.05.

Next, we wanted to understand why only a proportion of T cells differentiated into Tfh cells. Knowing that B cells play a role in their development and maintenance, we next wanted to understand if a small niche within secondary lymphoid organs limits Tfh cell differentiation and if these cells are terminally differentiated. Since nearly all BCL6^+^ Tfh cells in these mice were huCD2^+^ (**Figure 2C**), we sorted huCD2^+^ and huCD2^-^ CD4^+^ T cells from IL4^KN2/+^LAT^m/m^ mice and transferred them into LAT^-/-^ hosts (**Figure 6A**). Eight weeks after the transfer, these mice were analyzed. As shown in **Figure 6B**, in mice receiving huCD2^-^ T cells, there were 5% of T cells that were now actively secreting IL4 (huCD2^+^). These huCD2^+^ T cells converted from huCD2^-^ cells also expressed BCL6 (**Figures 6C–E**). On the other hand, in mice receiving huCD2^+^ T cells, 84% of the transferred T cells no longer were producing IL4 (huCD2^-^) and did not express BCL6. These data suggested that Tfh cells in LAT^m/m^ mice were not terminally differentiated and CD4^+^ T cells in these mice could gain or lose BCL6 expression.

### ICOS Is Not Required for Tfh Development in LATY136F Mice

Finally, we wanted to determine if other signaling pathways, in addition to ablated LAT-PLCγ1 interaction, was important for aberrant Tfh development. Our data in Fig.5 showed the importance of B cells in Tfh maintenance and BCL6 expression, we wanted to further understand which interactions between these cell types are also important. ICOS is a molecule that is expressed on T cells and is important for B and T cell interactions, GC formation, and antibody production (35–37). While it has been shown that ICOSL is not required for B cell activation in LAT^m/m^ mice (38), we wanted to examine the requirement of ICOS in the development of spontaneous Tfh and autoimmunity.

**Figure 7:**
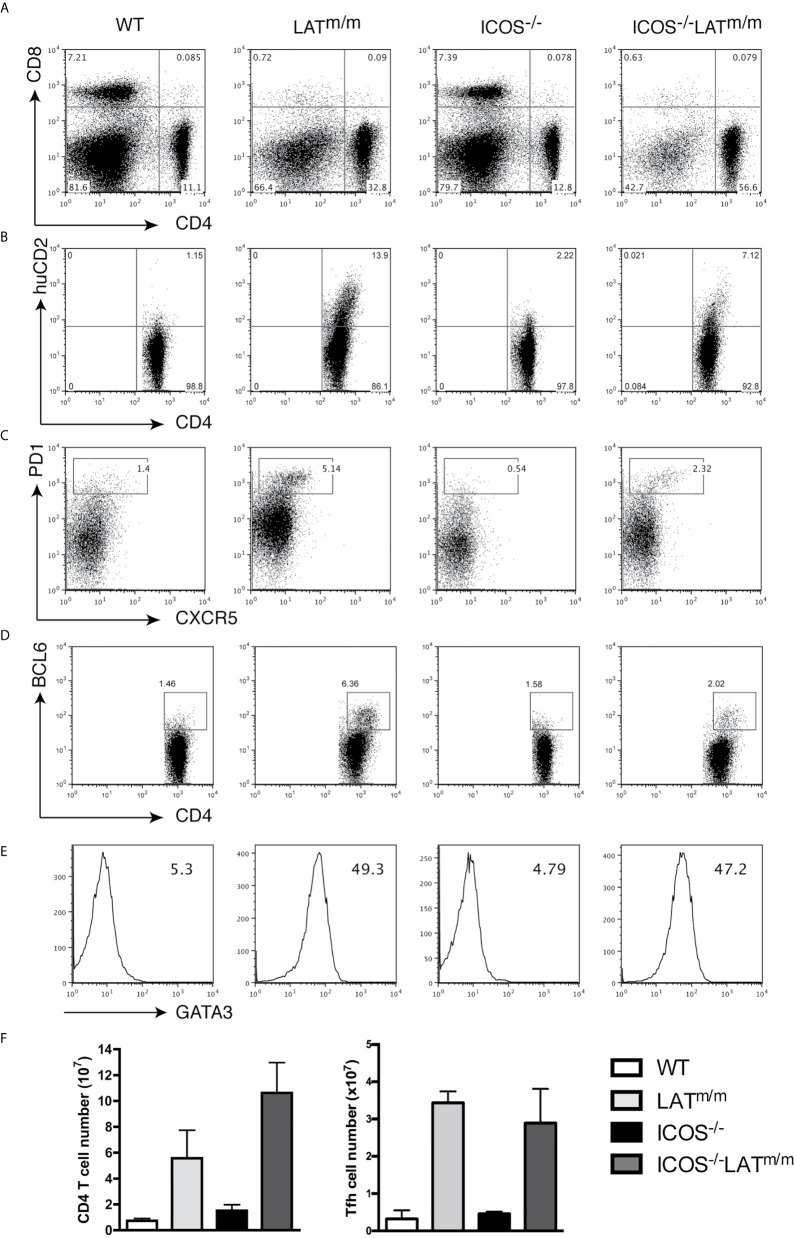
ICOS signaling is not required for Tfh development in LATY136F mice. WT, LAT^m/m^, ICOS^-/-^, and ICOS^-/-^LAT^m/m^ mice were all bred to IL4^KN2/+^ mice and were analyzed at 6-8 weeks of age. **(A)** CD4 and CD8 expression on splenocytes. **(B)** Surface staining of CD4 and huCD2. **(C)** Surface expression of CXCR5 and PD1. **(D)** Intracellular staining of BCL6. **(E)** GATA3 levels and gMFI. Cells were pre-gated on CD4^+^ T cells **(B–E)**. **(F)** Absolute numbers of CD4^+^ T cells and CD4^+^BCL6^+^ T cells. Data are representative of two individual experiments.

To this end, we crossed ICOS^-/-^ mice with LAT^m/m^ mice, and analyzed the development of Th2, Tfh, and the LATY136F autoimmune syndrome at 6-8 weeks of age. All mice were also IL4^KN2/+^ so that IL4 production could be accessed. LAT-mediated hyperproliferation was not affected by ICOS deficiency. Intriguingly, ICOS^-/-^LAT^m/m^ mice had increased percentages and numbers of CD4^+^ T cells compared to LAT^m/m^ mice (**Figures 7A, F**). However, LAT^m/m^ mice had higher percentages of huCD2^+^ IL4 producing, PD1^+^CXCR5^+^, and BCL6^+^ T cells than in ICOS^-/-^LAT^m/m^ mice (**Figures 7B–D**). CD4^+^ T cells in both LAT^m/m^ and ICOS^-/-^LAT^m/m^ mice expressed similarly high levels of GATA3 (**Figure 7E**). Therefore, while the percentage of Tfh cells appeared to be diminished in ICOS^-/-^LAT^m/m^ mice, the overall number of BCL6^+^ T cells was similar between LAT^m/m^ and ICOS^-/-^LAT^m/m^ mice (**Figure 7F**). These results indicated that ICOS is not required for spontaneous Tfh development in LAT^m/m^ mice.

## Discussion

Studies using LATY136F mice have established that tonic LAT-PLCγ1 signaling is important for regulating Th2 differentiation and IL4 production (15, 16). CD4^+^ T cells in the spleens and lymph nodes of these mice express high levels of GATA3 and produce IL4 upon PMA and ionomycin stimulation *in vitro*. Here, we showed that only a small proportion of these cells actually produced IL4 *in vivo*. These IL4 producing cells resembled Tfh cells, expressing BLC6, CXCR5, PD1, and ICOS. These cells were Blimp1^lo^ and SAP^hi^, and had elevated levels of IL21 mRNA. These Tfh cells, rather than Th2 cells, were found within B cell GC zones, and were actively producing IL4, similar to normal Tfh cells as described in previous studies (3).

Since BLC6 was identified as the master regulator of Tfh differentiation, many studies have tried to understand how BCL6 expression is regulated. Here we show that the LAT-PLCγ1 signaling axis is important for suppression of BCL6 expression in naïve T cells. Using an inducible deletion system, our studies showed that at two weeks after deletion of a wildtype LAT allele, naïve T cells with only a mutant LAT allele that was unable to bind PLCγ1 upregulated both BCL6 and GATA3. While our results suggested that B cells were important for development of these Tfh cells in LAT^m/m^ mice, we clearly detected Tfh skewing prior to B cell activation and isotype switching in our inducible system. Thus, the spontaneous Tfh differentiation is likely not a consequence of B cell activation in these mice.

A previous study found that BCR-mediated MAPK activation resulted in BCL6 phosphorylation and degradation (39). Abolishing the PLCγ1 binding site of LAT in Jurkat T cell line results in defective calcium mobilization and ERK activation upon TCR stimulation (12). Additionally, studies from our lab show that while p38 and PI3K signaling pathways are enhanced at the steady state, very little ERK activation is detected in LAT^m/m^ T cells (20). These data support the hypothesis that perhaps similar to B cells, without LAT-PLCγ1-mediated MAPK signaling, BCL6 in LAT^m/m^ mice is no longer suppressed, leading to the development of spontaneous Tfh cells.

Our adoptive transfer studies of T cells into mice deficient in T cells or both T and B cells suggested an important role for B cells in the differentiation and maintenance of BLC6-expressing LATY136F T cells. ICOS signaling is important for both Tfh cell differentiation and Tfh localization through its upregulation of CXCR5 (40); however, our data showed that ICOS signaling was not required for spontaneous Tfh differentiation (**Figure 7**). Several other key pathways and interactions between T and B cells have also been implicated in Tfh differentiation, such as OX40, SLAM family members, and cytokines (IL6 and IL21) (2, 41). It is possible that these pathways may compensate for ICOS deficiency. In support of this possibility, our previous work show that LATY136F mutation activates the p38, AKT, and NF-κB pathways, resulting in production of IL6 (20), which might be important in the interplay between T cells and B cells in this setting. Even though ICOS deficiency did not change the number of Tfh cells, their percentage was reduced due to increased T cell expansion in these mice. How ICOS deficiency increased T cell expansion is not clear. ICOS is also expressed on a subset of Treg cells. It is known that ICOS deficiency can cause Foxp3 instability due to significant methylation of Foxp3 CNS2, resulting in a reduced number of FOXP3^+^ Tregs. In addition, ICOS signaling can promote the proliferation and survival of Tregs (42–44). On the other hand, the LATY136F mice do not have Treg cells (18) or have abnormal Treg cells that have much reduced Foxp3 expression (45). It is possible that there are still some residual T cells that are able to suppress T cell hyperproliferation in LATY136F mice. ICOS deficiency might further diminish their suppressive activity, causing more T cell expansion.

Interestingly, our data showed that both spontaneous Th2 and Tfh development occurred in LAT^m/m^ mice. In these mice, Tfh cells expressed high levels of both GATA3 and BCL6. Tfh cells within the lymph node was characterized as IL4 producers that are GATA3^-^BCL6^+^ (27). It has also been demonstrated that BCL6 represses GATA3 expression at the post-transcriptional level (26) and that overexpression of BCL6 in Th2 cultures results in downregulation of GATA3 (9). When we analyzed aged LAT^m/m^ mice (>3 months), we detected lower GATA3 levels in BCL6^+^ cells compared to BCL6^-^ cells (data not shown), suggesting that BCL6 does repress GATA3 expression over time in these mice. However, during the initial differentiation of these T helper cells, BCL6 was not able to suppress GATA3. The reason for this abnormality remains to be investigated.

Our adoptive transfer experiments indicated that Tfh cells in LAT^m/m^ mice were not terminally differentiated. BCL6^+^ cells could develop from a BCL6^-^GATA3^+^ population, and huCD2^+^BCL6^+^ cell could drop their BCL6 expression. One could argue that when huCD2^+^BCL6^+^ T cells were transferred into LAT^-/-^ mice, there were a small number of contaminated huCD2^-^ CD4^+^ T cells that had undergone rapid expansion, resulting in reduced percentage of huCD2^+^BCL6^+^ cells in these mice. While possible, it is less likely to be the case. Unlike normal naïve T cells that can undergo huge homeostatic proliferation in a lymphopenic environment, the LATY136F T cells can only undergo slow expansion due to their defective TCR signaling pathway (45). As our data indicated that B cells were important in Tfh differentiation and maintenance, it is more likely that the constant engagement between T and B cells is required for maintaining their Tfh phenotype. When huCD2^+^BCL6^+^ T cells leave the germinal center or do not interact with B cells, they may lose their Tfh signature.

To conclude, our data indicated that LAT-PLCγ1 signaling is important in modulating Th2 and Tfh differentiation by repressing BCL6 expression in naïve T cells. While our study indicated the importance of the LAT-PLCγ1 signaling pathway in Tfh differentiation, it remains to be determined whether this abnormal Tfh differentiation indeed causes the lymphoproliferative syndrome. Further work will be needed to determine whether depletion of this subset of T cells can prevent T cells from uncontrolled expansion and ameliorate the autoimmune syndrome. Understanding the role of TCR signaling during development of autoimmunity may provide additional therapeutic targets to repress abnormal Tfh cells and the development of autoreactive B cells.

## Data Availability Statement

The original contributions presented in the study are included in the article/supplementary material. Further inquiries can be directed to the corresponding author.

## Ethics Statement

The animal study was reviewed and approved by Duke University IACUC.

## Author Contributions

SO performed experiments and wrote the manuscript. MZ performed experiments and edited the manuscript. WZ designed the experiments and edited the manuscript. All authors contributed to the article and approved the submitted version.

## Funding

This work was supported by National Institutes of Health Grant AI048674 and AI137756.

## Conflict of Interest

The authors declare that the research was conducted in the absence of any commercial or financial relationships that could be construed as a potential conflict of interest.
